# Scanning laser optical tomography resolves developmental neurotoxic effects on pioneer neurons

**DOI:** 10.1038/s41598-020-59562-7

**Published:** 2020-02-14

**Authors:** Karsten Bode, Lena Nolte, Hannes Kamin, Michael Desens, Arthur Ulmann, Gregor A. Bergmann, Philine Betker, Jennifer Reitmeier, Tammo Ripken, Michael Stern, Heiko Meyer, Gerd Bicker

**Affiliations:** 1 University of Veterinary Medicine Hannover, Institute of Physiology and Cell Biology, Bischofsholer Damm 15/102, 30173 Hannover, Germany; 2 Laser Zentrum Hannover e.V., Industrial and Biomedical Optics Department, D-30419 Hannover, Germany

**Keywords:** 3-D reconstruction, Neuroscience

## Abstract

Developmental neurotoxic compounds impair the developing human nervous system at lower doses than those affecting adults. Standardized test methods for assessing developmental neurotoxicity (DNT) require the use of high numbers of laboratory animals. Here, we use a novel assay that is based on the development of an intact insect embryo in serum-free culture. Neural pathways in the leg of embryonic locusts are established by a pair of afferent pioneer neurons, extending axons along a well-defined pathway to the central nervous system. After exposure to test chemicals, we analyze pioneer neuron shape with conventional fluorescence microscopy and compare it to 3D images, obtained by scanning laser optical tomography (SLOT) and processed by a segmentation algorithm. The segmented SLOT images resolve the 3D structure of the pioneers, recognize pathfinding defects and are thus advantageous for detecting DNT-positive compounds. The defects in axon elongation and pathfinding of pioneer axons caused by two DNT-positive reference compounds (methylmercury chloride; sodium(meta)arsenite) are compared to the biochemically measured general viability of the embryo. Using conventional fluorescence microscopy to establish concentration-response curves of axon elongation, we show that this assay identifies methylmercury chloride and the pro-apoptotic compound staurosporine as developmental neurotoxicants.

## Introduction

The developing fetal and juvenile human brain is more sensitive to exposure to industrial chemicals than the adult. Investigations of developmental neurotoxicity (DNT) deal with any adverse effects on the structure or function of the nervous system during gestational- or lactation-periods^1^. DNT positive chemicals pose a serious threat to the health of our children, because maldevelopment of the brain will eventually result in behavioral abnormalities, such as attention deficit, hyperactivity, and autism spectrum disorder^2,3^. Since there is growing concern that the increase of those neurologic defects may, at least in part, be caused by a cocktail of DNT positive compounds used in our daily life, there is now an urgent demand for risk assessment of industrial chemicals.

Until 2017, only 13 different substances have been identified as developmental neurotoxicants in humans by epidemiological approaches. In contrast, tens of thousands industrial compounds have not been studied so far and remain uncertain^2,4^. To evaluate these potentially toxic compounds large numbers of vertebrates over long testing periods are needed. Due to limitations of traditional animal testing by guidelines of the OECD, alternative assays have to be developed. Alternative *in vitro* testing methods focus on key aspects in brain development that can be readily quantified in cell culture assays. They include, for example, measurements of cell viability, cell proliferation, neurite extension, development of neurochemical phenotype, and electrical activity in randomly formed neural network as toxicological endpoints^5–8^. However, there are also non-mammalian organism based models such as zebrafish, *Caenorhabditis elegans*, and planarians^9–11^, displaying molecular developmental and functional aspects, which are conserved during the evolution. A recent review about alternative models for chemical assessment of DNT pointed out that these assays provide several advantages such as small size, short generation time, easy handling in the laboratory^8^.

An essential aspect of brain development is the anatomical wiring of neuronal processes. To form a functional nervous system, axons and dendrites have to navigate precisely through a complex microenvironment^12^. The tips of these growing processes are termed growth cones, which respond to extracellular chemotactic guidance cues, such as for example proteins of the semaphorin family^13^. One developmental strategy used by vertebrate and invertebrate nervous systems is to differentiate a special set of transient neurons whose axons pioneer the first fiber tracts. Axons, which develop later, follow these pathways already laid down. This navigational strategy is beautifully exemplified in locust embryos where the early axonal pathways in the peripheral nervous system are laid down by easily identifiable pioneer neurons^14^. For example, the neural pathways in the limb are set up by specific pairs of peripheral pioneer neurons that traverse the route from their origin near the tip of each appendage to the central nervous system (CNS), when distances are short^14,15^. These pioneer neurons have provided a useful model system for the study of a precise set of pathfinding processes during growth cone navigation. The cell bodies derive from the embryonic epithelium of the body appendage, remain in the periphery, and extend an axon that fasciculates with the axon of the sibling pioneer. Pathfinding seems to involve selective adhesion of the growth cones to substrate bound guidance cues provided by the epithelial cells and partly by recognition of guidepost cells^15^. A band of transmembrane semaphorin-1-expressing epithelial cells provides a pathway for the axons from the dorsal to the ventral side of the developing limb bud^13,16^. In addition, the epithelial cells of the limb bud secrete two gradients of the diffusible semaphorin-2a-molecule that orient the pioneer axons away from the periphery towards the CNS^17^.

A member of the semaphorin molecule family is also a key player in mammalian cortex formation. A gradient of semaphorin-3A is responsible for axonal and dendritic outgrowth pattern of pyramidal neurons during brain development^18^. Due to conservation of those guidance molecules between vertebrates and invertebrates, the pioneer neurons of the locust embryo may provide a useful model for examining blocking effects of neurotoxic chemicals on axonal outgrowth and navigation. We recently devised an intact embryo culture system in which defects in axonal elongation caused by exposure to chemicals are quantified in concentration-response curves^19^. The comparison of these curves to the biochemically measured general viability of the embryo allows for an evaluation of the DNT potential of chemicals, with regard to the endpoint axon elongation. On their pathway towards the CNS the developing pioneer axons show stereotypic changes in outgrowth direction^15^. To quantify both axonal extension and correct pathfinding, it would be advantageous to obtain three-dimensional images of the pioneers.

Scanning laser optical tomography (SLOT) is a relatively fast 3D-imaging technology that effectively detects absorption and fluorescence in transparent biological samples of mesoscopic size^20^. This technique is an advancement of classical optical projection tomography (OPT)^21^, which acquires projection images during rotation of the specimen at multiple equidistant angles. The 3D information is then obtained by reconstruction methods, such as the filtered back projection approach. In an improved laser based setup, SLOT optimizes signal collection efficiency especially at low signal levels^20,22,23^. To obtain mechanical stability during the rotation and optical clearing, it is advantageous to embed the specimen using the CRISTAL (Curing Resin-Infiltrated Sample for Transparent Analysis with Light) procedure, which clears the sample optically and embeds it in a rigid resin simultaneously without significant deformation as shown by Kellner *et al*.^22^.

Two of the well characterized DNT-positive reference compounds targeting animals and humans are methylated mercury and arsenic^4^. Here, we incubate intact locust embryos with the developmental toxicants methylmercuric chloride and sodium(meta)arsenite to test for their effects on pioneer neuron elongation and pathfinding. We analyze the resulting pioneer neuron outgrowth morphology with conventional fluorescence microscopy and compare it to 3D images, obtained by the SLOT technology and processed by a segmentation algorithm. In addition, we measure the acute toxicity of the respective compounds on the metabolic activity of embryo. The combined results demonstrate the potential of the SLOT imaging approach for resolving the 3D structure of the pioneers, the ability to quantitatively recognize pathfinding defects in the segmented images and to detect effects of established DNT-positive compounds in an alternative test assay.

Using the quantification method of Bergmann *et al*.^19^ for conventional fluorescence microscopy images, we generate concentration-response curves of axon elongation and determine the acute toxicity both for methylmercury chloride and the alkaloid staurosporine. Our assay identifies not only methylmercury chloride, but also the pro-apoptotic compound staurosporine as developmental neurotoxicants.

## Results

### Clearing and segmentation of SLOT images

**Figure 1:**
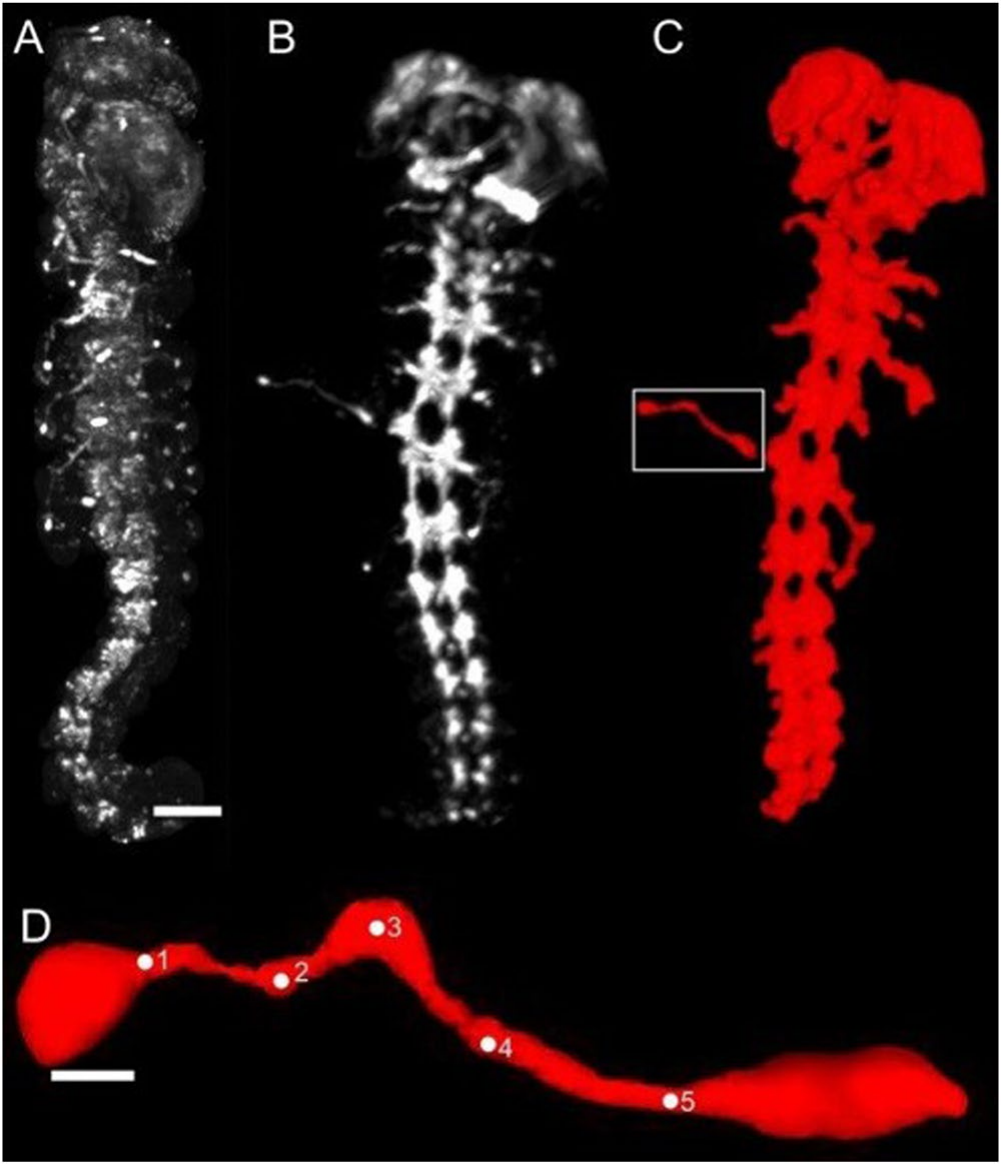
Clearing and segmentation of a locust embryo. To minimize light scattering from opaque tissue environment, embryos had to be cleared for SLOT imaging. Picture (**A**) displays an embryo cleared with glycerol (scale bar: 200 µm). In contrast, (**B**) shows an embryo cleared using the CRISTAL procedure as described by Kellner *et al*.^22^. The resulting rigid resin block bearing the cleared embryo is glued to a rotational axis, which can be incorporated into the SLOT system. The full projection data obtained by scanning the whole embryo is reconstructed and finally segmented in (**C**). Our focus is on the pioneer neurons, located in the hind legs as it is shown in (**D**) (white box in **C**). For 3D-length measurement, the axon is defined by setting 5 points along the pathway to the CNS to generate absolute length data (scale bar: 25 µm).

A widely-used method for the selective labeling of insect neurons is immunocytochemical staining with an antibody against the horseradish peroxidase (HRP) epitope^24,25^. For complete optical 3D imaging of the embryos after immunocytochemical labeling, the samples needed to be cleared to reduce light scattering by the tissue. Initially, liquid clearing in a series of glycerol was tested (Fig. 1A). The image shows the cell bodies of the pioneer neurons at the tip of the limb buds. The liquid clearing method required mounting of the embryos in a pivoted glass capillary, which could result in imaging artifacts due to movements of the embryo during image acquisition. Using the CRISTAL procedure^22^, the embryo could be cleared and embedded in a rigid resin block within the same step (Fig. 1B). The embedded sample was directly connected to the rotation motor of the SLOT setup. The embedding of the sample inside the solid resin reduced motion artifacts of the sample during rotation compared to solvent-based imaging techniques. Additionally, the CRISTAL procedure achieved a better clearing, resulting in less scattering and increased contrast between fluorescent nervous system and the remaining body tissue. Fluorescence intensity profiles transecting the legs resulted in a signal to background ration of 0.53 for CRISTAL and a lower ratio of 0.33 for glycerol-cleared embryos at the position of the pioneer axon. In Fig. 1B, both the ventral nerve cord of the central and the pioneer pathways of the peripheral nervous system are clearly resolved. This is especially apparent for one of the metathoracic legs which, for better visualization, was artificially stretched outside prior to fixation.

To quantify and segment the SLOT data, the semi-automated algorithm of the open source software ITK-SNAP was chosen^26^. The segmented binary image of the axon was subsequently displayed using the 3D viewer (https://imagej.nih.gov/ij/plugins/3d-viewer/) of ImageJ^27^ (Fig. 1C). Inside the 3D viewer, points were placed on the surface of the segmented axons (Fig. 1D). After extraction of the 3D coordinates of these points, the total length of the axon could be calculated by the Pythagorean quadruple. Rotating 3D views of the entire embryo (supplementary Fig. S1) and the segmented metathoracic pioneer neurons (supplementary Fig. S2) are presented in the supplement. Segmentation of the SLOT data of a complete embryo (Fig. 1C) resulted in a total tissue volume of 0.033 µl.

### Testing for DNT in locust embryos

**Figure 2:**
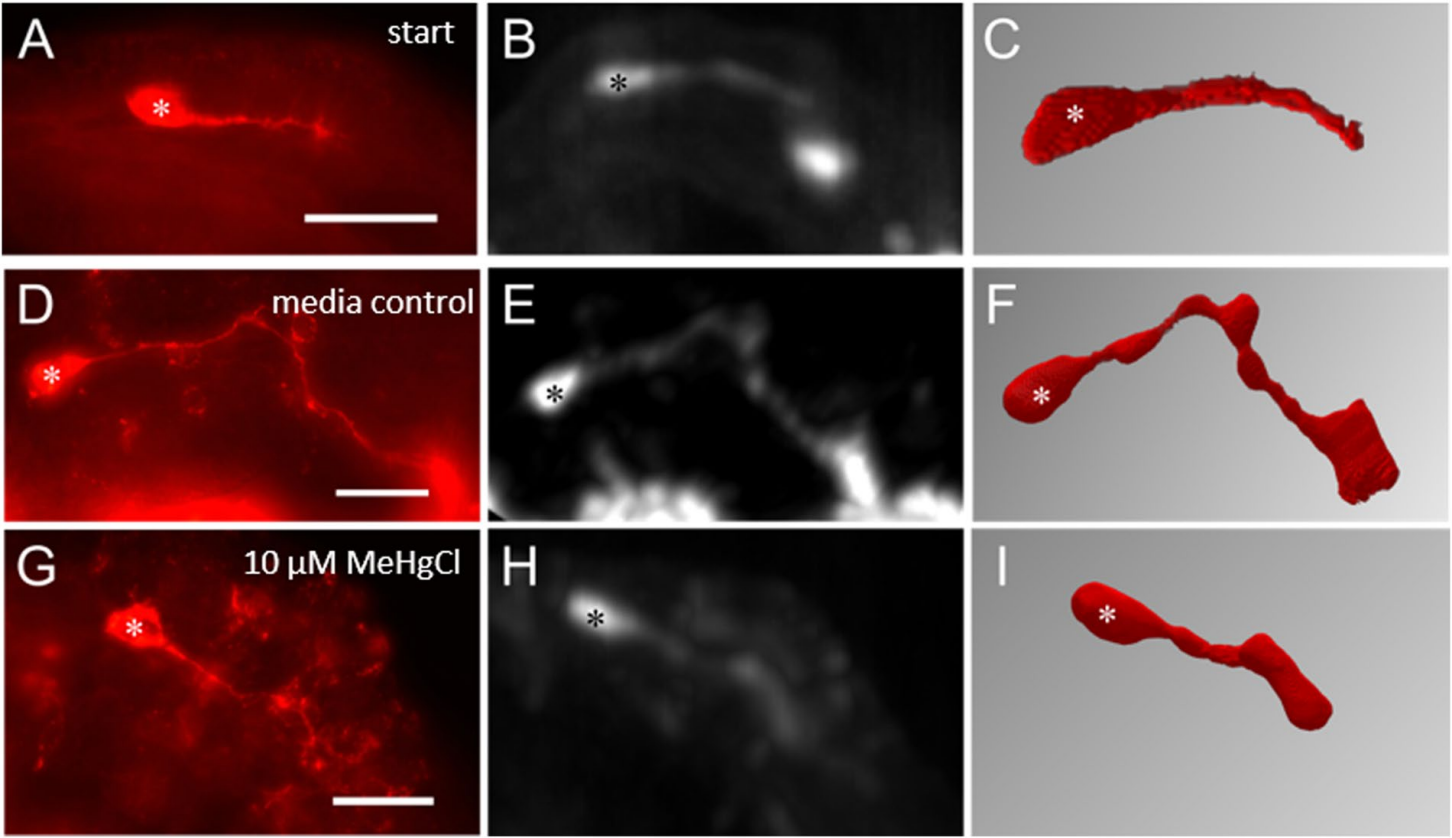
Incubation experiments for DNT testing with 10 µM MeHgCl. Pictures (**A**–**C)** show examples for start groups that were immediately fixed with paraformaldehyde. (**D**–**F**) represent media controls without test compound. The last row (**G**–**I**) depicts embryos treated with 10 µM MeHgCl. In this single range finding experiment, 10 µM MeHgCl led to no significant reduction of axon length compared to the media control group, neither by conventional fluorescence microscopy (left column) nor SLOT (middle column and right column: segmented data) technique. Asterisks are used to highlight cell bodies of pioneers. Scale bars: 50 µm. Measurement of axon length from SLOT data (**C**): 57 µm; (**F**): 181 µm; (**I**): 77 µm. Numbers of individual single measurements for conventional fluorescence microscopy A (n = 6), D (n = 20), G (n = 40) and SLOT B–C (n = 4), E–F (n = 15), H–I (n = 19).

One of the most thoroughly investigated DNT compounds is methylmercury. In a single range finding experiment, the test group was exposed to 10 µM MeHgCl for 18 hours. Using conventional fluorescence microscopy and the scoring scheme of Bergmann *et al*.^19^, we detected reductions of axon extension of the pioneer neurons in a number of specimens. Figure 2A shows the immediately fixed start situation (n = 6) after dissecting the embryo out of the egg. Compared to the embryos of the media control group (n = 20) (Fig. 2D), pioneer axons of MeHgCl-treated embryos were slightly shorter (n = 40) (Fig. 2G). However, because the experiment was only performed once in this range finding experiment, the number of evaluated embryos revealed no significant effect on the elongation score in the statistical test.

To test whether the absolute axon length determined by the SLOT imaging procedure was affected, these embryos were re-embedded using CRISTAL (Fig. 2B,E,H). Similar to the results of conventional fluorescence microscopy, segmented axons (Fig. 2C,F,I) of treated embryos (start n = 4; media controls n = 15; 10 µM MeHgCl n = 19) showed a reduction of axon extension when compared to the media control group. However, this effect was again statistically not significant.

**Figure 3:**
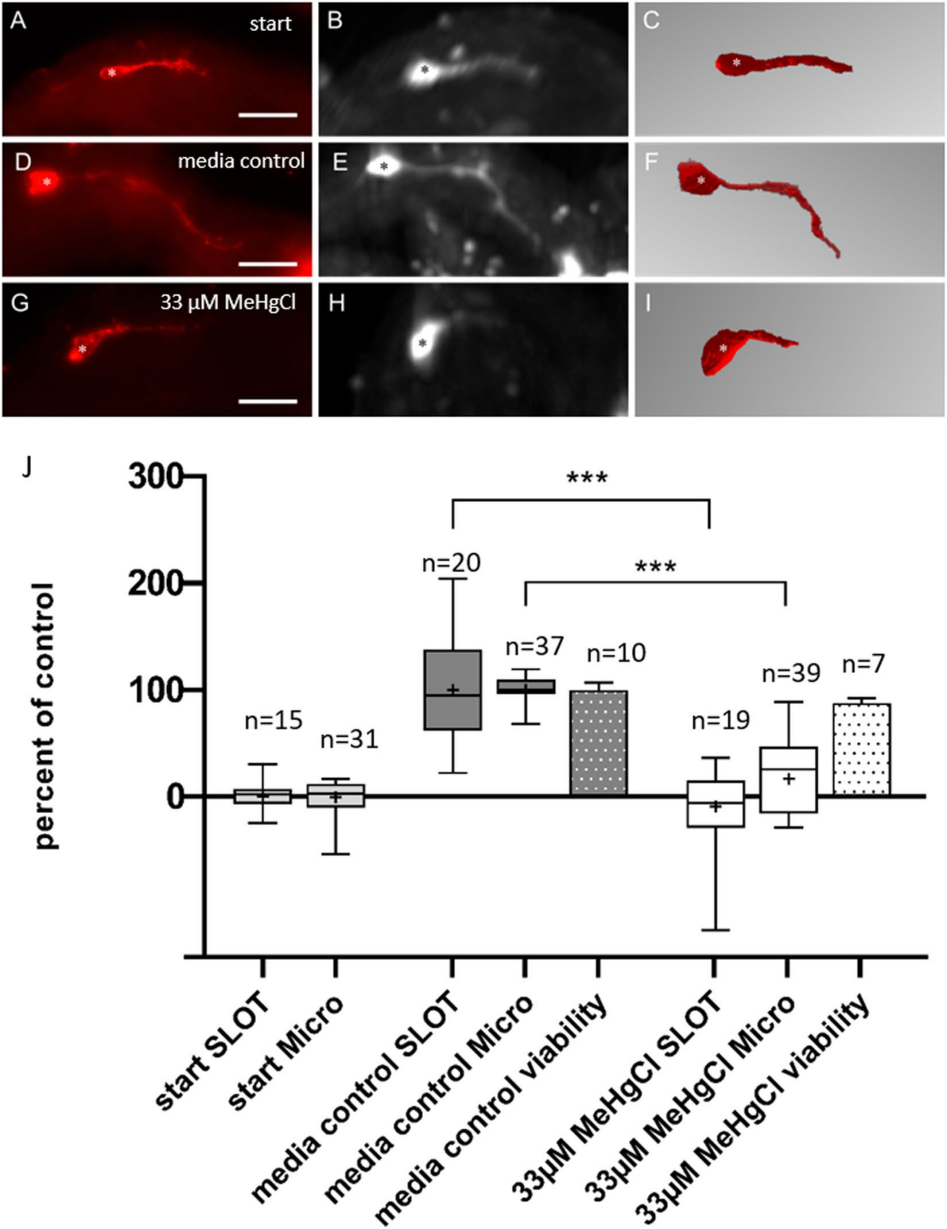
Comparison of conventional fluorescence microscopy and SLOT for DNT detection in locust embryos. Locust embryos of the same egg pod (technical replicates) were divided into three different groups (start, media control, and test group). The start group comprised embryos immediately fixed with paraformaldehyde in the beginning of the experiment. (**A**–**C**) Embryos incubated in media without any test compound are referred to as media control group (**D**–**F**), whereas embryos that were incubated with 33 µM MeHgCl belong to the test compound group. (**G**–**I**) Measurement of axon length from SLOT data (**C**) 94 µm; (**F**) 139 µm; (**I**) 42 µm. Biological replicates were pooled and normalized to their corresponding media control. To obtain the length of pioneer axons, data of all embryos were adjusted to their start group. In panel **J**, pioneer axon lengths of three independent experiments were pooled, normalized and investigated for their axon length during the absence or presence of the test compound (33 µM MeHgCl). Both conventional microscopy via fluorescence microscope (left column) and the SLOT technique (middle column and right column: segmented data) detected a significant reduction in axon length compared to the media control group. In contrast, viability measured by a resazurin assay was not affected after incubation with 33 µM MeHgCl (dotted bars). Asterisks are used to highlight cell bodies of pioneers. Statistical analysis were done by using Kruskal-Wallis test combined post hoc with multiple comparisons Dunn’s test. Scale bars: 50 µm. n = number of evaluated pioneer neurons. The middle line in the box-plots refers to the median and the + sign to the mean.

In a series of three independent experiments, we increased the concentration to 33 µM MeHgCl. Conventional fluorescence microscopy revealed different lengths for axon extension between the three groups (start group, media control group and test compound group, Fig. 3A,D,G). Start groups (n = 31) extended their axons to 48 ± 4 (mean ± SEM) of the elongation score. Media control group axons (n = 37) reached 89 ± 1 (mean ± SEM) of the scoring scheme. Following normalization, these values were set to 0% (start groups) and 100% (media control groups). After incubation in 33 µM MeHgCl (n = 39) pioneer axons showed a reduction of outgrowth to 55 ± 3 (mean ± SEM) of the elongation score. After normalization, this is equivalent to 17% axon extension of control (Fig. 3J).

Similar results were obtained, when the same embryos were examined by SLOT. The start groups (n = 15), extended their axons to 80 ± 19 µm (mean ± SEM) (Fig. 3B,C). After 18 hours of incubation, axons of culture media controls (n = 20) reached 182 ± 24 µm (mean ± SEM) (Fig. 3E,F). The treatment with 33 µM MeHgCl (n = 19) reduced axon length to 51 ± 9 µm (mean ± SEM) (Fig. 3H,I), shorter than at start (Fig. 3J). The histogram of Fig. 3J shows normalized data of three independent experiments, revealing significant differences of axon length between media control groups and test compound groups by both conventional fluorescence microscopy (p < 0.001) and SLOT (p < 0.001). To distinguish between general cytotoxic and developmental neurotoxic effects on axonal extension, we measured the viability of the embryo by a resazurin assay (alamarBlue^TM^). In this biochemical assay, viability was not significantly affected. At 33 µM (when neurite outgrowth was completely blocked) the viability still reached 88% of the media control group, indicating a developmental neurotoxic effect of MeHgCl on axonal extension (Fig. 3J).

**Figure 4:**
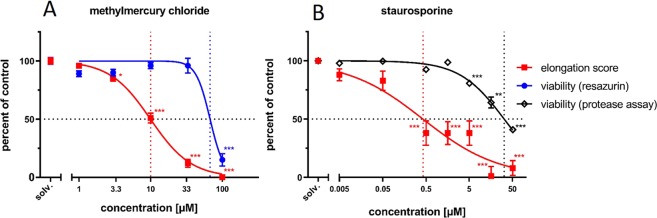
Concentration-response curves of methylmercury chloride (**A**) and staurosporine. (**B**) Each concentration-response curve is the average (mean ± SEM) of at least three independent experiments. Data points were normalized to their media controls and start values, respectively. Viability was measured by either a resazurin reduction assay (blue curve) or a dead-cell protease assay (black curve). Neurite lengths of pioneer neurons were determined by the elongation score and subsequently compared to media controls (red curve). Statistical analysis was done by a Kruskal-Wallis and multiple comparisons Dunn’s test (*p < 0.05, **p < 0.01, ***p < 0.001). Dotted lines indicate the half-maximal inhibitory concentration (IC 50). Numbers of individual single measurements for (**A**) n = 62 (solv. represents the media control group), n = 35 (1 µM), n = 46 (3.3 µM), n = 56 (10 µM), n = 36 (33 µM), n = 9 (100 µM). (**B**) n = 66 (solv. represents the media control group), n = 30 (0.005 µM), n = 15 (0.05 µM), n = 24 (0.5 µM), n = 21 (1.58 µM), n = 29 (5 µM), n = 23 (15.8 µM), n = 18 (50 µM).

To quantify the developmental neurotoxic effect of MeHgCl on pioneer axon extension in our test system, we generated concentration-response curves using the established scoring scheme^19^ and the resazurin assay (Fig. 4A). In this series of experiments, we incubated more than 141 embryos, resulting in a total number of n = 244 single axon length measurements. The quantitative evaluation showed that MeHgCl inhibits axon growth in a dose-dependent manner. At 3.3 µM MeHgCl, axon elongation was already reduced to 85 ± 4% (mean ± SEM) compared to media controls (p = 0.0404). Incubation with 10 µM MeHgCl caused a reduction to 50% of media controls (p < 0.001), resulting in an IC 50 of 9.96 µM. In contrast, the viability decreased only significantly at the highest test concentration. The IC 50 of the viability curve was estimated to 67.53 µM.

Next, we generated a concentration-response curve (n = 226) for the pro-apoptotic compound staurosporine which has been listed as possible DNT positive compound^28^ (Fig. 4B). At concentrations of 0.5 µM, staurosporine reduced axon elongation about 50% compared to media controls. Since apoptosis is often an energy-dependent process^29^ that might result in increased resazurin reduction, we used an alternative type of viability assay, based on dead-cell protease detection (CytoTox-Fluor^TM^ Cytotoxicity Assay). At a concentration of 5 µM, staurosporine significantly reduced the viability (p < 0.001), with an estimated IC 50 of 31.68 µM. Based on the large difference (63-fold) of the IC 50 for the elongation score and the viability, our pioneer neuron assay classifies staurosporine as developmental toxicant.

**Figure 5:**
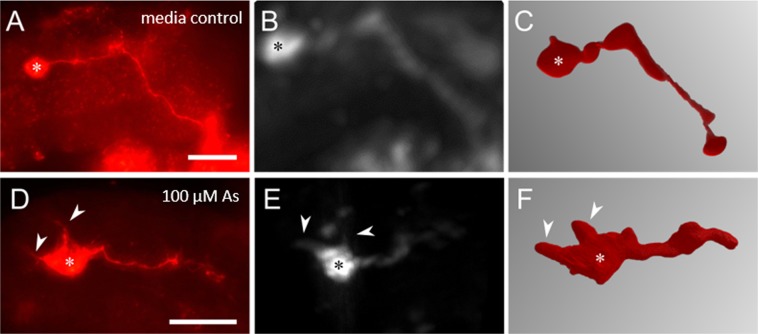
Investigation of pioneers for axonal defects after treatment with arsenic. Embryos exposed to 100 µM sodium(meta) arsenite were characterized by abnormal axon outgrowth behavior (arrows) of pioneer neurons shown in (**D**–**F**) in comparison to media controls. (**A**–**C**) Both visualization methods (right column: conventional fluorescence microscopy; SLOT: middle column and right column: segmented data) were capable to resolve errors during establishment of the pathway to the CNS. Asterisks are used for cell body marking. Scale bars: 50 µm. Measurement of axon length from SLOT data (**C)**: 130 µm; (**F**): 46 µm.

Epidemiological studies indicate that arsenic is also a developmental neurotoxicant for humans^30^. At a concentration of 100 µM, incubation with sodium(meta)arsenite resulted in abnormal pioneer axon outgrowth and navigation. The cell bodies of insect neurons typically give rise to a single neurite which, in case of the pioneers develops as the extending axon (Fig. 5A–C). As shown in Fig. 5D–F, the cell bodies of the pioneer neurons generate several neurites which were recognizable by conventional fluorescence microscopy and SLOT. Moreover, there were navigational defects during the establishment of the pathway from cell bodies to the central nervous system (Fig. 5D–F). These data show that the insect assay can reveal defects both in axon extension and navigation.

## Discussion

With respect to axonal outgrowth, intact locust embryos can provide an alternative to animal experiments in DNT testing^19^. There are several advantages of this system. Up to 50 embryos of the same age can be dissected from a single egg pod allowing for multiple replicates of experiments and establishment of concentration-response curves. Exposure to test compounds occurs in an intact embryo in which the generation of neurons, establishment of early neural pathways and differentiation of neural phenotypes has been described at single cell resolution^31–33^. Meanwhile the complete *Locusta migratoria* genome including transcriptome and methylome data has been published^34^ allowing targeted transcript knockdown via RNAi^35^. Moreover, in some cases, such as for example the leg pioneer neurons, cellular and molecular mechanisms for growth cone navigation have been elucidated in remarkable detail^15,16,36^. As shown by antibody blocking experiments, growth cone navigation depends on membrane-bound or molecular gradients of diffusible semaphorin cues^17^. The semaphorin protein family, first discovered in the locust embryo^16,17^, is conserved in vertebrates^37^ including mammalians, where semaphorins play an important role in brain cortex formation^18^. This evolutionary consideration raises the possibility that the pioneer axon assay may be used to uncover compounds interfering with semaphorin-mediated signal transduction also in vertebrate neural development.

*In vivo* studies suffer from problems of undefined exposure parameters such as actual concentrations in the tissue of interest due to diffusion barriers and metabolic turnover. Since the early embryo is not covered by a chitineous cuticle, it is, similar to cell based assays, permeable to small molecule ligands and even antibodies. Moreover, equivalents of a mammalian placental barrier, a blood-brain barrier, and efficient metabolisation of test compounds by a maternal liver are not present. These properties permit the blocking experiments and chemical manipulation of intracellular signaling pathways^38^, responsible for pioneer neuron navigation. We calculated from SLOT data a total volume of 0.033 µl for an embryo staged at 35% of development. This value provides an approximation of the magnitude of the tissue volume available for metabolism. In the standard incubation procedure, two embryos were cultured together in a single well in a volume of 200 µl of L-15 medium containing the test compounds. We view it rather unlikely that approximately 0.066 µl of embryonic tissue will be able to metabolize or chelate large proportions of a certain test compound dissolved in the excess volume of 200 µl. Based on the permeability to small molecule ligands^38^, antibody blocking experiments^17^, and the consideration of the incubation volume, we surmise that the outgrowing pioneer neurons are exposed to effective concentrations of test compounds that closely match the concentrations presented in the graphs. All these features of the insect embryo allow us to address in our test system one of the key characteristics of a developing nervous system development: the establishment of a specific axonal pathway.

Chemical compounds interfering with calcium signaling, the cytoskeletal organization and the reference developmental neurotoxicant rotenone, were already classified as DNT positive in the axonal elongation assay using conventional fluorescence microscopy^19^. This assay identified endpoint specific inhibitors of calcium-dependent growth cone motility and general cytoskeletal inhibitors as specific effectors, compared to general cytotoxicity measurements, an essential requirement for a DNT assay^4^.

In this paper, we showed that MeHgCl inhibits pioneer axon elongation (Figs. 3 and 4A) in a concentration range where the viability of treated embryos was not affected (Figs. 3J and 4A). Thus, our insect assay classifies this compound as developmental neurotoxic. Other alternative DNT assays have also shown that methylmercury is a potent developmental neurotoxicant. It can interfere with several endpoints such as migration or differentiation in human Ntera2 cells^39^ or neurite outgrowth in PC12 cells at nanomolar levels^40^. Interestingly, neuropathological effects in humans, such as decreases in brain size, damage to cortex or behavioral alterations, could be seen at high-level exposure with estimated brain concentrations from 55 µM to 93 µM MeHg. In contrast, even lower brain concentrations could lead to morphological changes in animal experiments (<14 µM MeHg)^41,42^. This range of effective concentrations is in line with a detection threshold of 3.3 µM methylmercury and an IC 50 of 9.96 µM (Fig. 4A), using the scoring scheme of the conventional fluorescence microscopy assay^19^. To account for biological variability, it is evident that multiple separate experiments are required to derive a reliable estimate of the detection threshold and the IC 50 of the pioneer axon assay. Even though single specimens (Fig. 2F,I) showed an inhibitory effect on axon growth, a single range finding experiment was not sufficient to reveal statistically significant differences in axon length between media control and 10 µM MeHgCl-treated embryos. This applies both to the conventional fluorescence microscopy assay and SLOT imaging. Differences in the variability between groups (Fig. 3J) prepared for conventional fluorescence microscopy, which were later re-embedded for SLOT imaging are partly due to the technical issue that specimens have been lost in the CRISTAL procedure, accounting for the lower number of evaluated legs in Fig. 3J. Other contributing factors are the completely different methods of axon length quantification. The conventional assay relies on the scoring scheme that is based on the axon reaching a certain landmark visible in the fluorescence microscope. The SLOT imaging assay requires an image segmentation step in which faintly fluorescent parts of the thin axons may drop below detection threshold, resulting in a broader distribution of the axon lengths.

The zebrafish has emerged as promising vertebrate model for developmental neurotoxicity assessment^43^. A transcriptional analysis of the CNS and other organs of the 3-day-old zebrafish embryo demonstrated that sublethal concentrations of 0.3 µM MeHg induce tissue specific expression changes in genes linked to cellular stress responses, without affecting the overall structure of the developing brain^44^. The detection limit of our insect system is not sufficient for using axon extension as a bioassay to predict long-term toxicity of MeHg in environmental studies. Life cycle tests of selected fish models are about three orders of magnitude more sensitive^45,46^. However, our assay aims to detect and quantify specific developmental neurotoxicity of chemical compounds.

More than 200 million people are chronically exposed to arsenic which, apart from its carcinogenic and acute toxic effects, is classified as DNT-positive compound^2,30^. Experiments on Neuro2a cells, a mouse neuroblastoma cell line, also suggest that arsenic treatment reduces neurite outgrowth prior the onset of apoptosis^47^. Incubation of the locust embryos with 100 µM arsenic induced defects in axonal elongation, navigation, and the formation of aberrant neurites (Fig. 5). In comparison, anatomically observable alterations in neural development including malformation of the spinal cord and disordered motor axon projections required a two days arsenic exposure of zebrafish embryos at a concentration of 2 mM^48^. Here, the locust embryo assay proves more sensitive.

The pro-apoptotic alkaloid staurosporine is not an established DNT-positive compound, but has been listed as potential candidate in a systematic review on DNT testing methods^28^. Staurosporine acts mainly as a broad-spectrum protein kinase inhibitor^49^ and will most likely interfere with many cellular processes. After induction by staurosporine, the energy-dependent cellular degradation mechanism of apotosis will require time for completion of cell death. We assume that an assignment of staurosporine either as DNT-positive or negative compound will depend on the incubation time. The 63-fold difference of the IC 50 for the elongation score and the viability (Fig. 4c) classifies staurosporine as developmental toxicant under the incubation conditions of our pioneer neuron assay.

Quantification of neurite length is useful to discover chemical compounds which harm nervous system development. Apart from neurotoxicity assays, another potential application lies in the screening of drugs that enhance axonal outgrowth. In this respect, conventional 2D fluorescence microscopic examination of embryo whole mounts is rather unfavorable, because the viewing of the pioneer neurons is restricted through torsion or positioning of the curved limb bud. The 3D images generated by SLOT avoid these limitations and allow for an exact quantification of neurite length while the same established fluorescence based staining protocol is used. Furthermore, it provides improved collection efficiency, isotropic resolution and no shading effects compared to other fluorescence based 3D imaging techniques as optical projection tomography, confocal laser scanning microscopy or light sheet microscopy. As examples, the figures in the supplement show the 3D structure of the pioneer axons within the environment of surrounding tissue.

We compared the conventional fluorescence method^19^ with 3D imaging using the SLOT technology (Fig. 3J). Pioneer axons of three independent experiments were investigated for their neurite length during the absence or presence of the test compound (33 µM MeHgCl). Both conventional microscopy via fluorescence microscope and the SLOT technique detect a significant reduction in neurite length compared to the media control group. In contrast, viability, measured by a resazurin assay, is not affected after incubation with 33 µM MeHgCl (Fig. 3J, dotted bars). After exposure of the embryos to arsenic, the SLOT technique resolved also defects in axonal navigation and abnormal generation of neurites. Furthermore, the introduction of SLOT imaging could provide an additional step forward towards semi-automated DNT-detection in an alternative model system. In this methodological testing phase of the assay, we relied on setting manually a fluorescence intensity threshold such that the segmentation algorithm could reliably reconstruct the 3D image of the pioneer neurons. A further development of a fully automated image recognition tool would allow for a more rapid and completely unbiased scoring of developmental toxic effects on axonal navigation.

Many chemical compounds have not been investigated for their developmental neurotoxic potential, because current test methods for DNT potential require the use of high numbers of laboratory animals^50^. This lack of knowledge together with the consideration of the three Rs (replacement, refinement and reduction of animal experiments)^51^ produced an urgent demand on alternative assays that can reliably detect DNT-positive chemicals. The introduction of *in vitro* assays using rodent primary cells, human cell lines, stem/progenitor based-, and organoid systems^7,8,28,52,53^, has meanwhile generated considerable progress in the development of alternative test systems. However, at least to our knowledge, none of these assays has addressed axonal navigation of identified neuron types.

This paper presents an *ex vivo* systemic approach that addresses some of the complexity of the *in vivo* situation in a simple embryonic invertebrate preparation. We are now calibrating the assay against a battery of positive reference compounds with known DNT potential in humans and negative compounds, which are toxic, but have no specific DNT potential. Since our overall goal is the development of predictive *in situ* assays for identification of developmental neurotoxicity, we plan to monitor birth and death of the transient pioneers, but also of other sensory neurons at defined developmental stages. This will allow for the incorporation of toxicological endpoints for neurogenesis and apoptosis in our insect embryo assay. Insect preparations could contribute to reduce or replace animal experiments, when integrated in a battery of different DNT tests that assay for the various endpoints^8^. The combination of mesoscale invertebrate embryos with optical 3D imaging allows for complete registration of neuron morphology and thereby enables sensitive and reliable detection of axonal growth impairment.

## Materials and Methods

### Animals and preparation

The methods followed essentially the description by Bergmann *et al*.^19^. Embryos of *Locusta migratoria* were obtained from our own culture at the University of Veterinary Medicine Hannover. The locusts were raised in breeding cages at a temperature of 30 °C in a 12 hour light and dark cycle. Fertilized female locusts provided with moistened sand are able to lay up to 50 eggs in a single egg pod. 24 hours after laying the egg pods into sand, the pods were removed and subsequently stored at 32 °C in an incubator for 3 days. The pods were placed on moistened filter papers in a horizontal position, providing similar oxygen access to the humidified air. Then embryos were dissected in serum free Leibovitz L-15 medium containing 1% penicillin/streptomycin (Invitrogen). After removing the amnion membrane, only embryos staged to 32.5% of embryogenesis^54^ were used.

### Incubation and exposure to test compounds

For an individual experiment, embryos of a single egg pod were collected in pairs in 200 µl L15 (with solvent, as appropriate) in 48 well plates, in rows of at least 3 wells per concentration (technical replicates). Embryos were briefly washed in 200 µl test solution, and incubated in fresh test solution at 30 °C for 18 h. Each egg pod contributed to three groups of embryos that were essential for analyzing the developmental neurotoxicity potential of test compounds. Start groups: To quantify the length of neurites in the beginning of the experiment, about 5 embryos were immediately fixed for immunolabeling. Media control groups: The media control group comprised locust embryos cultured in L15 medium without test compound. Test compound groups: Embryos incubated with either methylmercury chloride (Sigma), staurosporine (Sigma) or arsenic (Sigma) for 18 hours. For creating concentration-response curves, each row contained three to five technical replicates, depending on the size of the egg pod. These technical replicates were incubated in different concentrations of the test compound. A concentration-response curve required at least five repeats from different experiments (biological replicates). After 18 hours of incubation with the test compound (with DMSO, if appropriate), the endpoint of viability was determined in a biochemical assay. In preliminary experiments, we confirmed that DMSO alone had no effect on general viability or neurite elongation at concentrations up to 1%. Final DMSO concentrations in test compound groups did not exceed 0.1%.

### Viability assays

The viability of locust embryos was measured by an alamarBlue^TM^ viability assay (Invitrogen). This assay is based on the ability of living cells to reduce resazurin into the fluorescent resorufin. The amount of fluorescent product is proportional to the number of living cells and was detected in a microplate reader (Tecan). After incubation of embryos with the test compound in a 48-well plate, the supernatant was removed. To avoid any cross-reactions between the test substance and the viability assay embryos were rinsed in L15 medium for 5 minutes. Embryo pairs were subsequently incubated in L15 medium including 5% of alamarBlue^TM^ assay for two hours at 30 °C. To obtain the background signal, a blank without any embryo was placed on the 48-well plate. Finally, the embryos were fixed with 4% paraformaldehyde for further immunofluorescence labeling analysis. Since apoptosis depends on cellular metabolic activity^29^, experiments with the pro-apoptotic compound staurosporine required an alternative viability assay (CytoTox-Fluor^TM^ Cytotoxicity Assay, Promega). This fluorescent assay measures the integrity of cell membranes after chemical exposure, using the supernatant. When chemicals disrupt membranes, the release of dead-cell proteases is detected via microplate reader. The released proteases cleave the fluorogenic peptide substrate (bis-AAF-R110). In living cells, the substrate is not able to pass the membrane, leading to no fluorescence signal. The number of released dead-cell proteases is inversely proportional to the viability of cells.

### Immunolabeling

All three groups of embryos were processed for immunofluorescence labeling. Embryos were fixed in 4% paraformaldehyde/PBS (Sigma) for 60 minutes, followed by permeabilization with 0.3% saponin (Sigma) in PBS with Triton-X100 (0.1%, Sigma, PBS-T) for 45 minutes. Then, the embryos were washed in PBS-T 0.1% for 3 times before they were blocked (30 minutes) with PBS-T 0.1% containing 5% normal rabbit serum (NRS, Linaris).

The Ti1-pioneer neurons were labeled with HRP antibodies that are able to detect a specific carbohydrate epitope expressed on the neuronal surface of insect neurons^24,25^. The antibody (goat-anti-HRP, Dianova) was diluted 1:2000 in blocking solution, containing PBS-T 0.1% and 5% NRS (Linaris). Next, the embryos were incubated with HRP antibodies over night at 4 °C.

Following the immunolabeling, preparations were washed three times for 10 minutes respectively, before they were incubated with the biotinylated secondary antibody (1:250, rabbit-anti-goat in blocking solution, Dianova) for one hour at room temperature (RT). After three additional washing steps for 10 minutes in PBS-T, the second antibody was detected by incubation for one hour at RT with Streptavidin-Cy3 (Sigma), diluted in PBS-T 0.1% (1:250).

### DAPI

**Figure 6:**
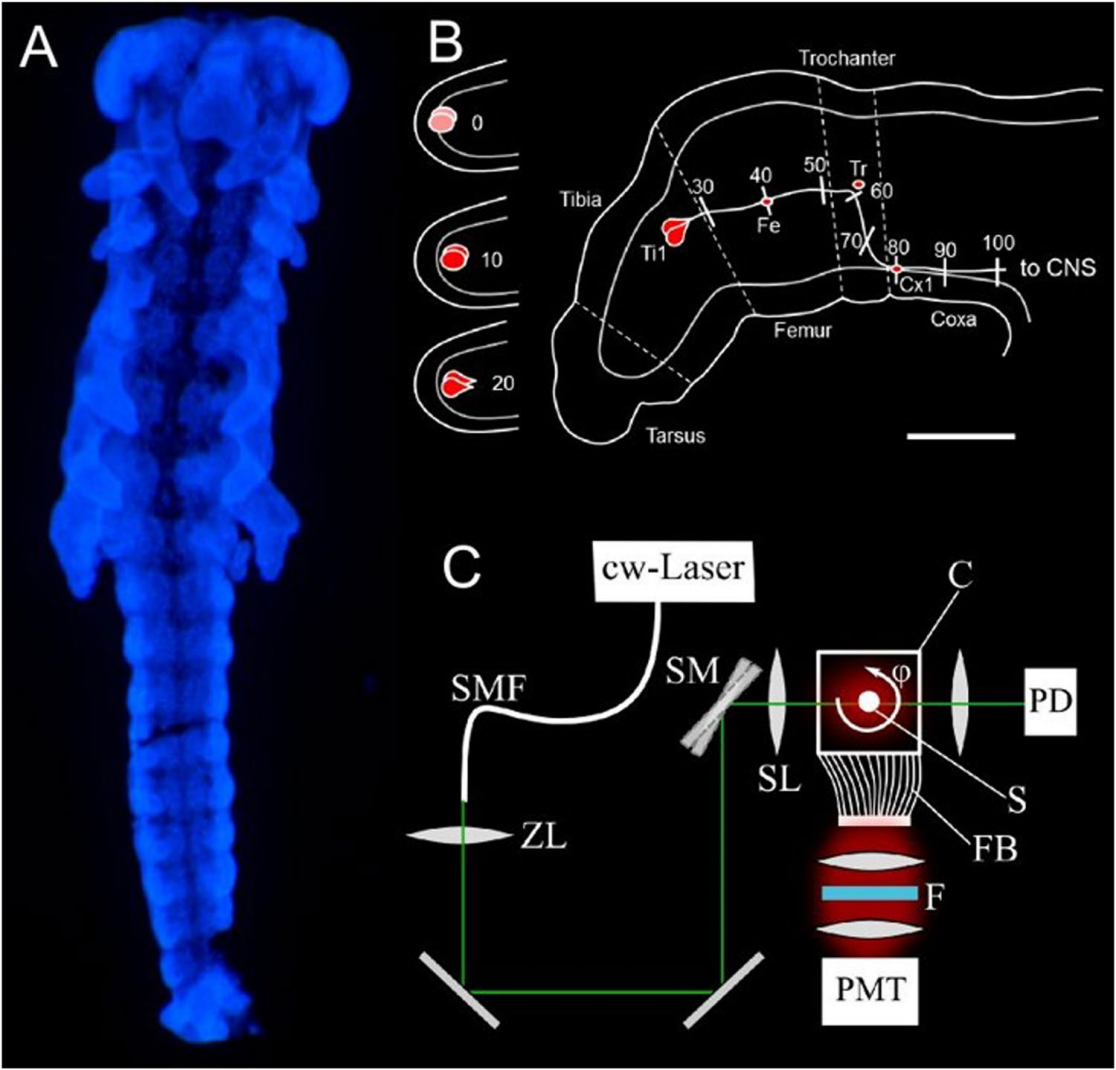
The locust embryo as a system for developmental neurotoxicity testing. Picture (**A)** depicts a locust embryo staged to 35% of embryonic development and labeled with DAPI for its cell nuclei. Figure (**B)** illustrates a schematic of a metathoracic limb bud. At this age, the complete pathway to the CNS is laid down. The pioneer siblings (Ti1) arise within the tibia segment (shown in red) and extend their axons stereotypically to the CNS, passing three guidepost cells (Fe, Tr, Cx1) on their way. The established pathway is divided into ten percent intervals from 0 (pioneers are not born or only faintly stained) to 100 (axons reached the CNS). Figure (**C)** displays the setup of the SLOT system, which is used for measuring pioneer length in a three-dimensional context. cw-Laser: continuous wave laser; SMF: single mode fiber; ZL: zoom lens; SM: scanning mirror; SL: scanning lens; C: cuvette; S: sample; ϕ: rotation angle; FB: fiber bundle; F: filter; PD: photo diode; PMT: photo multiplier tube.

While adding the secondary antibody linked to Streptavidin-Cy3, embryos were also incubated with DAPI (4.6-diamino-2-phenylindole, Sigma) for labeling of the cell nuclei (Fig. 6A). DAPI was diluted in PBS-T 0.1% (1:500, 0.1 µg/ml). Subsequently the embryos were rinsed again two times in PBS-T 0.1% and once in PBS 0.1% for each 10 minutes. All samples had to be cleared up for 30 minutes in Glycerol/PBS 1:1 and 10 minutes in Glycerol/PBS 9:1.

### Conventional fluorescence microscopy

For microscopic evaluation, embryos were mounted on glass slides between two spacers and covered with cover slip. Labeled pioneer neurons were visualized using a conventional fluorescence microscope (Carl Zeiss Axioskop) combined with Axiocam 506 color and the Compact Light Source HXP 120 V. Images were taken by the means of ZEN 2012 blue edition software version 1.1.2.0. Afterwards, images were stacked by ImageJ 1.51q software^27^ with the setting *max intensity*.

### Evaluation of axon outgrowth

To quantify the axonal length of Ti1 pioneer neurons after exposure to test compounds, the elongation score^19^ was determined using conventional fluorescence microscopy. For each leg bud, elongation of pioneer axons along their predefined pathway was scored between 0 and 100 according to the scheme depicted in Fig. 6B. This so-called elongation score rates the axonal outgrowth in relation to easily recognizable landmarks within the metathoracic leg, such as guidepost cells and segment boundaries. Pioneer neurons of both hindleg buds of each embryo develop independently. Thus, the axon elongation score of the pioneer axons of each leg bud is considered an individual measurement. From each individual measurement, the average elongation score of the start groups of this experiment was subtracted, to receive the elongation between start and end of the experiment. In a next step, each elongation value was normalized to the average elongation of the media control group of this experiment. This resulted in individual outgrowth values between 0% and 100% (or even above 100%, when a neuron had grown farther than the media control groups). Embryo viability measurements were also normalized to the average viabilities of the media control groups (after subtraction of the average blank values). Normalization allowed for pooling of data from different separate experiments, to reflect biological variability. Concentration-response curves were generated by fitting four parameter sigmoidal functions and IC 50 values were derived. The normalized data were analyzed by the Kruskal-Wallis test followed by Dunn’s multiple comparisons test (GraphPad Prism version 8.01 for Windows, GraphPad Software, La Jolla California USA, www.graphpad.com).

### Sample clearing for SLOT imaging

For initial tests, embryos were cleared in a glycerol series and the whole mounts were scanned in the SLOT setup using a glass capillary containing a mixture of 90% glycerol in PBS. To account for imaging artifacts (compare results section), the clearing method was changed from the liquid clearing approach to the CRISTAL procedure^22^. The embryos were infiltrated by a monomer (NOA 86, Norland Products), which matches in polymerized state the refractive index of the sample (n = 1.56). After polymerization of the UV-cured monomers, the embryos are entirely encapsulated within a transparent cylindrical solid body. This resin block was glued to the rotational axis of the SLOT setup to ensure mechanical stability during image acquisition. Details of the SLOT procedure using CRISTAL embedding were described by Kellner^22^.

### 3D visualization in SLOT

The principle of SLOT has been described in detail elsewhere^20,55^. Briefly, the light of a 520 nm laser diode (LD-520-50SG, Roithner Lasertechnik GmbH, continuous wave (cw) Laser) was coupled into a single mode fiber (SMF, P3-460, Thorlabs Inc, USA) to generate a Gaussian beam profile (Fig. 6C). The output of the single mode fiber was collimated and enlarged in diameter to 10 mm by a zoom telescope (ZL, H10Z1218MP, computar, CBC AMERICAS Corp., USA). The beam was subsequently directed on an x-y-scanning mirror system (ProSeries II Scan Head, Cambridge Technology, USA), which is positioned in the back focal plane of the telecentric F-Theta scanning lens (S4LFT0080/121, Sill Optics GmbH & Co.KG, Germany, focal length: 75 mm). This optical system resulted in a spatial resolution of 4 µm and a depth of field of 200 µm. This covered one-half of the locust embryo width. During a full rotation of the sample, all points of the sample would be in focus. For axon investigation, it was sufficient to position the respective axon in the focus. The embedded embryo was connected to a rotational stepping motor (M-060.PD, Physik Instrumente GmbH, Germany) and placed inside a cuvette. The cuvette was filled with a silicon oil (INDOMET TETRA, Indomet e.K., Germany), matching the refractive index of the CRISTAL polymer (n = 1.55) to avoid diffraction at the sample-liquid-interface. The distance between scanning lens and embryo was adjusted to the focal length of the scanning lens, to obtain a sharp image of the embryo. The fluorescence of the Cy3 labeled embryo was collected from the bottom of the cuvette by a fiber bundle, which fit the shape of the cuvette and directed the light on a band pass filter (565/24 BP, F37-565, AHF, Germany) and subsequently on a photomultiplier tube (R3896, Hamamatsu Photonics K.K., Japan). Simultaneously, transmitted light was directed on a photodiode (PDA36A, Thorlabs Inc, USA) to measure the absorbance inside the sample. Projection images were generated pixel by pixel with a pixel dwell time of approximately 8 µs. The number of acquired pixels in x- and y-direction was adjusted to the orientation of the imaged embryo individually, resulting in an approximate pixel size of 2 µm (typical number of pixels: 500 × 500 px²). Respectively, the number of projections (angle increments) was also adjusted individually with a typical value of 800 projections for a full rotation of the sample. SLOT imaging of a complete locust embryo required an approximate measurement time of 45 minutes. In this paper, only fluorescence data generated by the PMT was used. The full projection data set was reconstructed using a filtered back projection algorithm from the open source software IMOD^56^. 3 D images of pioneer neurons were evaluated for axon lengths. Normalization of data and statistical analysis was performed as described above.

## Supplementary information

Supplementary Information.

Supplementary Figure S1.

Supplementary Figure S2.

## Data Availability

Data used in this study are available on reasonable request.
