# Supplementary material for: Scanning laser optical tomography resolves developmental neurotoxic effects on pioneer neurons
Source: Sci Rep. 2020 Feb 14;10:2641. doi: 10.1038/s41598-020-59562-7 (PMC7021824; doi:10.1038/s41598-020-59562-7)
Supplement: Supplementary file 1 — Supplementary Information. [file 41598_2020_59562_MOESM1_ESM.doc]

**Scanning laser optical tomography resolves developmental neurotoxic effects on pioneer neurons**

Karsten Bode*^1^, Lena Nolte*^2^, Hannes Kamin^2^, Michael Desens^2^, Arthur Ulmann^1^, Gregor A. Bergmann^1^, Philine Betker^1^, Jennifer Reitmeier^1^, Tammo Ripken^2^, Michael Stern^1^, Heiko Meyer^2^ & Gerd Bicker^1^

^1^ University of Veterinary Medicine Hannover, Institute of

Physiology and Cell Biology, Bischofsholer Damm 15/102, 30173 Hannover, Germany

^2^ Laser Zentrum Hannover e.V., Industrial and Biomedical Optics Department, Germany

*^1^ *^2^*These authors contributed equally to this work*

**Supplementary figures**

**Supplementary figure S1: 3D reconstruction of a whole mount locust embryo.** The rotating 3D view of a locust embryo resolves the central- and peripheral nervous system (red) using SLOT imaging and the segmentation algorithm. The cell bodies of pioneer neurons are located in the distal tip of each limb bud, extending their axons to the central nervous system. For better visualization of pioneer neurons, one metathoracic leg was artificially stretched outside during fixation.

**Supplementary figure S2: 3D reconstruction of pioneer neurons in limb bud.** The rotating 3D view shows the pair of parallel running Ti1 pioneer neurons (red) within a surface rendering of the metathoracic limb bud. The two distal cell bodies appear fused. The reconstruction based on SLOT imaging and the segmentation algorithm shows the characteristic pathway of pioneer neurons growing into the central nervous system.
