# Supplementary figures and images for: Scanning laser optical tomography resolves developmental neurotoxic effects on pioneer neurons
Source: Sci Rep. 2020 Feb 14;10:2641. doi: 10.1038/s41598-020-59562-7 (PMC7021824; doi:10.1038/s41598-020-59562-7)

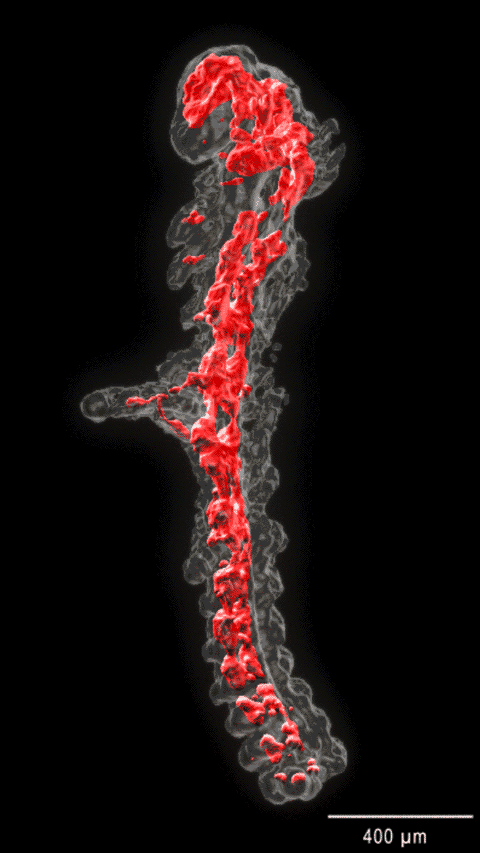

Supplement: Supplementary file 2 — Supplementary Figure S1. [file 41598_2020_59562_MOESM2_ESM.zip › Supplementary Figure S1.gif]

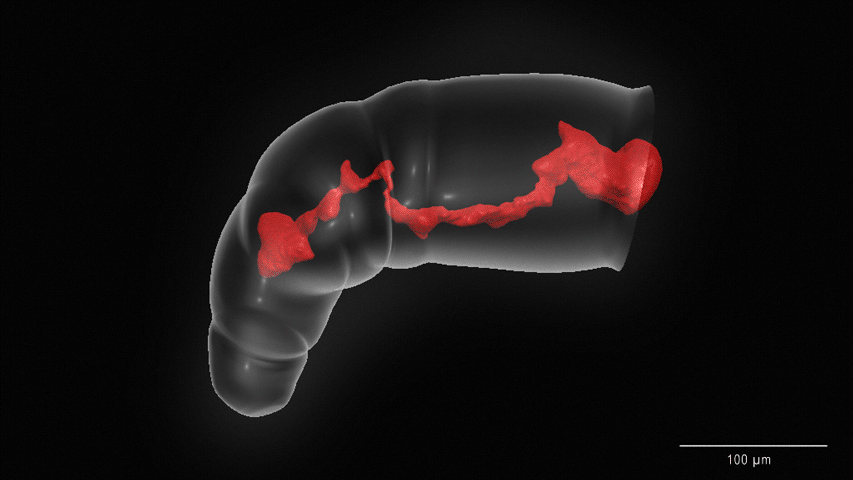

Supplement: Supplementary file 3 — Supplementary Figure S2. [file 41598_2020_59562_MOESM3_ESM.zip › Supplementary Figure S2.gif]
